# Micropyramid Array Bimodal Electronic Skin for Intelligent Material and Surface Shape Perception Based on Capacitive Sensing

**DOI:** 10.1002/advs.202305528

**Published:** 2023-11-29

**Authors:** Hongsen Niu, Xiao Wei, Hao Li, Feifei Yin, Wenxiao Wang, Ryun‐Sang Seong, Young Kee Shin, Zhao Yao, Yang Li, Eun‐Seong Kim, Nam‐Young Kim

**Affiliations:** ^1^ RFIC Centre Department of Electronics Engineering NDAC Centre Kwangwoon University Seoul 01897 South Korea; ^2^ School of Information Science and Engineering University of Jinan Jinan 250022 China; ^3^ Department of Molecular Medicine and Biopharmaceutical Sciences Seoul National University Seoul 08826 South Korea; ^4^ College of Micro & Nano Technology Qingdao University Qingdao 266071 China; ^5^ School of Microelectronics Shandong University Jinan 250101 China

**Keywords:** 3D printing, electronic skin, fringing effect, intelligent perception, iontronic effect, machine learning

## Abstract

Developing electronic skins (e‐skins) that are comparable to or even beyond human tactile perception holds significant importance in advancing the process of intellectualization. In this context, a machine‐learning‐motivated micropyramid array bimodal (MAB) e‐skin based on capacitive sensing is reported, which enables spatial mapping applications based on bimodal sensing (proximity and pressure) implemented via fringing and iontronic effects, such as contactless measurement of 3D objects and contact recognition of Braille letters. Benefiting from the iontronic effect and single‐micropyramid structure, the MAB e‐skin in pressure mode yields impressive features: a maximum sensitivity of 655.3 kPa^−1^ (below 0.5 kPa), a linear sensitivity of 327.9 kPa^−1^ (0.5–15 kPa), and an ultralow limit of detection of 0.2 Pa. With the assistance of multilayer perceptron and convolutional neural network, the MAB e‐skin can accurately perceive 6 materials and 10 surface shapes based on the training and learning using the collected datasets from proximity and pressure modes, thus allowing it to achieve the precise perception of different objects within one proximity‐pressure cycle. The development of this MAB e‐skin opens a new avenue for robotic skin and the expansion of advanced applications.

## Graphical abstract

In this work, a novel micropyramid array bimodal (MAB) electronic skin (e‐skin) based on the fringing effect and iontronic effect is proposed for proximity and pressure sensing. Motivated by the machine learning algorithms, the proposed MAB e‐skin can accurately perceive different material species and surface shape types within one proximity‐pressure cycle.

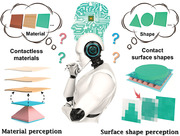

## 1 Introduction

Tactile perception refers to the process of converting tactile information obtained through the human skin into bioelectric signals, which are then carried by afferent nerves to the central nervous system in the brain. Following complex analysis, processing, and learning, deeper information that is difficult to measure directly can be extracted.^[^ ^1^, ^2^, ^3^ ^]^ Inspired by this, researchers have developed a range of flexible electronic devices known as electronic skins (e‐skins) that are extensively studied for their ability to convert tactile information into electrical signals.^[^ ^4^, ^5^ ^]^ Up to now, the acquisitions of various types of physical information (e.g., pressure, temperature, humidity) have been successfully achieved through the contact or contactless interaction of e‐skins with objects based on resistive, capacitive, triboelectric, and piezoelectric principles, and have been applied in flexible robots,^[^ ^6^, ^7^ ^]^ intelligent prosthetics,^[^ ^8^, ^9^ ^]^ and metaverse.^[^ ^10^, ^11^ ^]^ It is worth noting that the realization of the aforementioned e‐skin and its application scenarios requires excellent sensing performance to be ensured, and the introduction of micro‐nano structures effectively solves this problem. There are currently many highly competitive methods for the preparation of micro‐nano structures. For instance, the template transfer method based on anodic aluminum oxide (AAO) can be used for the preparation of nanoscale cone‐ or pillar‐shape structures.^[^ ^12^ ^]^ However, AAO templates are a family of self‐organized/assembled hydrophobic ceramic materials that can be precisely controlled to form only certain types of hollow nanostructures, making it challenging to achieve sharp tips and specific designs of micro‐/nano‐scale pyramid arrays. In contrast, photolithography and deep reactive‐ion etching methods can more effectively prepare micro‐/nano‐scale pyramid arrays with different morphologies by adjusting experimental parameters. Chen et al.^[^ ^13^ ^]^ employed a combination of photolithography and etching techniques to process inverted micropyramid Si templates (feature size: 4.5 µm) and assemble high‐performance e‐skin based on the micropyramid array. Despite its advantages in achieving micro‐nano structures with high precision and controllability, the preparation process is complex, time‐consuming, and uneconomical, rendering it unsuitable for large‐scale production. In addition, with the integration of artificial intelligence (AI) technologies, e‐skin also possesses the unique tactile perception ability of humans.^[^ ^14^, ^15^, ^16^, ^17^, ^18^ ^]^ For instance, Sundaram et al.^[^ ^19^ ^]^ developed a scalable tactile glove that can confer function to perceive object types and estimate object weights through the post‐processing of tactile information with deep convolutional neural networks. Zhu et al.^[^ ^20^ ^]^ reported a robotic hand that combines tactile sensing information with machine learning (ML) and can precisely perceive different shapes and sizes of various objects. Tee et al.^[^ ^21^ ^]^ enabled the rapid perception and classification of object textures by combining a tactile resistive annularly cracked e‐skin with a neural network model for analyzing sensing signals. These studies made a breakthrough in achieving a tactile perception comparable to that of human beings. Nonetheless, certain functions that exceed human tactile perception, such as material perception, wherein the surface is flat and difficult to distinguish among objects, remain extremely challenging. Despite various technologies that have been developed in recent years to perceive material species, such as ultrasonic, thermal conductivity, and triboelectric, and have made significant progress,^[^ ^20^, ^22^, ^23^, ^24^, ^25^ ^]^ combining two or more perception technologies to extract multiple characteristics of materials for gradually achieving a broader range of material perception in the future remains extremely challenging. Consequently, novel and reliable material perception technologies are desperately awaited to further enrich and bridge the gaps in the field of material perception.

On the basis of the coupling of the fringing effect and the mutual capacitance effect, different materials generate varying capacitance responses when they approach capacitive e‐skin. This diversity in response is attributed to differences in the dielectric constants among materials.^[^ ^26^ ^]^ When placed near the capacitive e‐skin sequentially, the disturbance of the fringe electric field caused by them and the distribution of the electric field lines are different,^[^ ^27^, ^28^ ^]^ thus allowing for the accurate perception of multiple materials with the help of the mutual capacitance effect, making the capacitive e‐skin a powerful tool for inferring material properties. Additionally, precise perception of an object often requires the coordination of multiple attributes, particularly the material species and surface shape types, which are essential elements for perception. However, most currently developed devices with object perception abilities require the utilization of either two distinct e‐skins or at least two distinct readout mechanisms,^[^ ^23^, ^29^, ^30^ ^]^ that inevitably increase the amount of space occupied by the sensing architecture and thus reducing the density of e‐skins that can be integrated into the system. Fortunately, a single capacitive e‐skin can realize material perception in proximity mode while also perceiving the surface shape of an object through spatial pressure mapping in the form of an e‐skin array; this enables the fusion of two different mechanisms in a single device and effectively solves the limitations of integration density, but this has rarely been studied. Hence, the material and surface shape of the objects can be perceived in turn according to the capacitance response in both proximity and pressure modes through a fixed detector. Pursuing research on material and surface shape perception based on the capacitive sensing mechanism will certainly play a pivotal role in complementing and promoting the advancement of the intelligent perception field, and will comprehensively boost the continuous evolution of e‐skin technology in AI.

**Figure 1:**
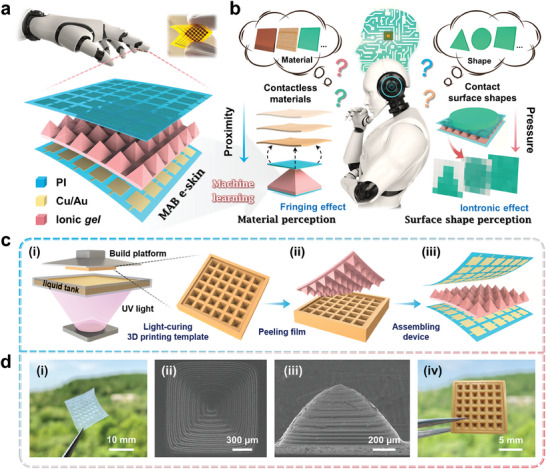
Construction drawing of the intelligent perception based on MAB e‐skin. a) Structure and photograph of the MAB e‐skin. b) Schematic of the intelligent material and surface shape perception of the MAB e‐skin based on capacitive sensing (fringing and iontronic effects). c) Fabrication process of the MAB e‐skin: i) light‐curing 3D printing anti‐micropyramid array template, ii) template‐transferred micropyramid array, and iii) assembling device. d) Photograph i) and SEM images (top‐view ii) and cross‐sectional iii)) of ionic gel with micropyramid array, and photograph iv) of the anti‐micropyramid array template.

Herein, a micropyramid array bimodal (MAB) e‐skin based on capacitive sensing is designed, consisting of 6 × 6 electrode arrays and 36 single‐micropyramid structure ionic gel prepared by light‐curing three‐dimensional (3D) printing technology (**Figure** **1**a). Motivated by the ML algorithms, we propose a novel method to perceive the material and surface shape of different objects by exploiting the fringing effect of the single‐micropyramid e‐skin (a sensing unit in MAB e‐skin) and the iontronic effect of MAB e‐skin, respectively (Figure 1b). Additionally, the multiphysics modeling of the fringing and iontronic effects is conducted using COMSOL simulation to validate systematically the bimodal sensing mechanism. The proposed MAB e‐skin in pressure mode features a maximum sensitivity of 655.3 kPa^−1^ for pressures below 0.5 kPa, a linear response corresponding to a sensitivity of 327.9 kPa^−1^ within the pressure range of 0.5–15 kPa, and an ultralow limit of detection (0.2 Pa). Given its bimodal sensing, spatial mapping applications are explored and demonstrated, including contactless measurements of 3D objects and contact recognition of Braille letters. Furthermore, the key concept of our device is to infer the material species and surface shape types based on the capacitance response generated by the object and MAB e‐skin in proximity and pressure modes sequentially, thus achieving the purpose of accurately perceiving different objects. As a proof of concept, it is demonstrated that MAB e‐skin can accurately perceive 6 materials and 10 surface shapes by training and learning the collected response curves (proximity mode) and 36‐channel response‐pressure mapping (pressure mode) datasets with the assistance of a multilayer perceptron (MLP) and convolutional neural network (CNN), thus allowing it to perceive precisely different objects within one proximity‐pressure cycle. Furthermore, ML‐based data processing minimizes environmental interference, substantially improving the perception accuracy to 92.3% and 97.2%, respectively. The proposed MAB e‐skin combined with ML will open up reliable new paths for the intellectualization of robotic skin, thus enabling it to truly possess tactile perception abilities comparable to or even better than those of human beings.

## 2 Results and Discussion

### 2.1 Structural Design and Characterization

Figure 1a illustrates the structure and optical image of the proposed MAB e‐skin, which has an overall area of 1 × 1 cm^2^ (6 × 6 matrix, 36 single‐micropyramid e‐skin). The e‐skin consists of PI/Cu/Au electrode arrays and poly(vinylidene fluoride‐co‐hexafluoropropylene)/1‐ethyl‐methylimidazolium bis(trifluoromethylsulfonyl)imide (PVDF‐HFP/[EMIM][TFSI]) ionic gel. The fringing effect unique to this typical “sandwich” structure, along with the iontronic effect constructed by ionic gel, serve as the cornerstones for achieving proximity and pressure sensing, respectively (Figure 1b). The ionic gel is synthesized by mixing [EMIM][TFSI] into the matrix of PVDF‐HFP (Figure S1, Supporting Information), which is an elastomer with a relatively high dielectric constant of 9 to facilitate ion delocalization. Furthermore, the formation of ion‐electron pairs at the interface between the ionic gel and the electrode greatly enhances the capacitance response under tiny pressure, thereby improving pressure sensitivity, while largely minimizing the impact of parasitic capacitance. The fabrication process of the MAB e‐skin is depicted in Figure 1c, where all components are made from economically viable materials, and can using patterned by simple micro‐processing technologies (see “Experimental Section” for fabrication details). Figure 1d(i–iii) and Figure S2a,b (Supporting Information) show the optical image, top‐view scanning electron microscope (SEM) images, and cross‐sectional SEM images of the ionic gel with the micropyramid array. The micropyramid structures are observed to be intact, featuring areas that decrease uniformly from bottom to top. The single‐micropyramid structure will serve as the intermediate layer within one of the sensing units in the MAB e‐skin, thus providing the necessary guarantee for the high‐sensitivity linear response of pressure and the performance consistency among single‐micropyramid e‐skins. In this study, the designed template is constructed using light‐curing 3D printing technology to achieve various microstructure patterns considering its low cost in fabricating microstructures with uniform morphology that can be used in mass production in the future; this structure will then be transferred to the ionic gel. The surface morphology of the anti‐micropyramid array template is shown in Figure 1d(iv) and Figure S3 (Supporting Information).

### 2.2 Proximity Sensing Characteristics

**Figure 2:**
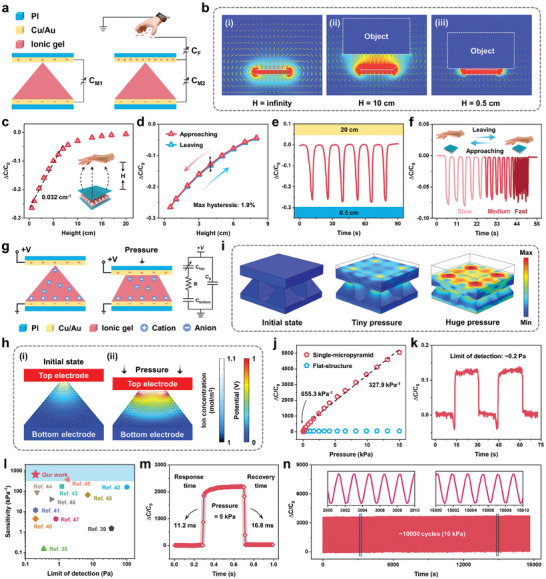
Characterizing the proximity and pressure sensing performances of the single‐micropyramid e‐skin. a) Sensing mechanism based on fringing effect in proximity mode. b) COMSOL simulation results of the potential distribution of an object near the single‐micropyramid e‐skin at different heights. c) Relative capacitance variations in response to an approaching palm. d) Capacitance response versus distance during a palm approaching and leaving cycle. e) Approaching and leaving repeatability test (0.5–20 cm). f) Capacitance response changes to the vertical movement of the palm at different frequencies. g) Sensing mechanism based on iontronic effect in pressure mode. h) COMSOL FEA simulation of the potential distribution and ion concentration gradient with/without pressure. i) Stress distribution of COMSOL FEA simulation results for the single‐micropyramid e‐skin at various pressures. j) Pressure‐dependent relative capacitance changes in the e‐skins with single‐micropyramid and flat‐structure. k) LOD of ≈0.2 Pa (vertically approaching and horizontal moving away). l) Comparison of the sensitivity and LOD of the proposed e‐skin with recently reported capacitive e‐skin. m) Response and recovery time at a pressure of 5 kPa. n) Stability tested over 10 000 cycles at a high pressure of 10 kPa.

Due to the bimodal sensing of proximity and pressure of MAB e‐skin, we will introduce them separately to demonstrate their excellent sensing capabilities in a more thorough and organized manner. Using the single‐micropyramid e‐skin as an example, to verify its proximity sensing characteristics, the palm is used as an object close to the e‐skin, and the vertical distances between the palm and the e‐skin are calibrated using a ruler. As depicted in **Figure** **2**a, the proximity sensing mechanism of the e‐skin originates from the disturbance of the fringing electric field within the parallel‐plate capacitor, i.e., the fringing effect. This mode involves two types of capacitances: the mutual capacitance (*C*_M_) between the top and bottom electrodes (measured parameter in the entire process), and the fringing capacitance (*C*_F_) between the palm and the top electrode (grounded through the human body).^[^ ^31^, ^32^ ^]^ Because the human body acts as a grounded electrode, the electric field lines of the capacitor complete the path to ground through the human body (electric field lines are shunted), thus reducing the electric field strength associated with the capacitor plate and decreasing the charge stored in the capacitor.^[^ ^33^ ^]^ As the distance between the palm and the top electrode decreases, charges flow out from the two electrodes, thus resulting in an increase in *C*_F_ and a decrease in *C*_M_. Figure 2b(i) shows the simulated potential distribution near the e‐skin using COMSOL when the uncharged object is placed at an infinite distance; the arrow indicates the divergent electric field with uneven distribution at the edge of the e‐skin. As the object is equivalent to another ground electrode, the electric field lines between the device electrode and the object cause a shunt phenomenon when the object approaches (Figure 2b(ii,iii)), further verifying the *C*_F_ and *C*_M_ variation rules. In addition, once the object contacts the e‐skin (H = 0 cm), the electric field between the object and the device will disappear completely (Figure S5, Supporting Information). As a result, charges on the electrode do not flow out, thus making *C*_F_ approach 0 and *C*_M_ increase to its initial value (object at infinity).

According to Figure 2c, the single‐micropyramid e‐skin exhibits a large detection range (0‐20 cm) in proximity mode, with a maximum sensitivity of 0.032 cm^−1^ within a range of less than 7 cm, and the sensitivity is not affected by temperature and humidity (Figure S6, Supporting Information). The sensitivity decreases with distance from the e‐skin, which is due to the fact that the electric field lines of the *C*_M_ are sparse at locations far from the e‐skin, thus resulting in fewer electric field lines passing through the human body into the earth and causing a smaller change in capacitance. As depicted in Figure 2d, two capacitance response curves are highly overlapped during the palm approach and leave the e‐skin (vertical distance: 0–8 cm), indicating good reversibility and negligible hysteresis (maximum: 1.9%, height: 4 cm). To evaluate the cycle repeatability of the e‐skin, the approach and leave cycles within a vertical distance in the range of 0.5–20 cm are tested, and a reproducible and stable response is displayed as shown in Figure 2e. Moreover, the capacitance response of the palm when approaching and leaving the e‐skin at different frequencies is also tested (Figure 2f); the e‐skin exhibits a stable capacitance responses during both low‐ and medium‐frequency movements. However, the capacitance response in the high‐frequency movement is quite different and is caused by the inability to control precisely the distance between the palm and the e‐skin within a certain range owing the fast‐moving palm. Moreover, the response and recovery times in the high‐frequency movement are 44.8 and 33.6 ms (limited by the palm movement frequency) (Figure S7, Supporting Information).

### 2.3 Pressure Sensing Characteristics

The sensing characteristics of MAB e‐skin in pressure mode are described below. Taking the single‐micropyramid e‐skin as an example, In Figure 2g, the basic principle and equivalent circuit diagram of iontronic sensing are illustrated, showing positive and negative ion pairs distributed throughout the ionic gel. Unlike typical parallel‐plate capacitive sensing (see Figure S8 in the Supporting Information and the corresponding explanation), iontronic sensing primarily relies on an ion‐electron capacitive interface, known as the electrical double layer (EDL) effect. The emergence of the EDL causes the electrons in the electrode to attract and gather at nanometer distances with the oppositely charged ions in the ionic gel, thus forming numerous small capacitors in parallel, which significantly improves the specific capacitance of the e‐skin.^[^ ^34^, ^35^ ^]^ Figure 2h illustrates the use of COMSOL finite element analysis (FEA) to model the EDL effect to simulate the ion movement and distribution in the aforementioned process. In the initial state, the contact area between the tip of the micropyramid structure and the top electrode is extremely limited; thus, only a small portion of the anions in the ionic gel gather towards the top electrode to form the EDL (Figure 2h(i)). When pressure is applied, the top electrode and the micropyramid structure of the ionic gel are squeezed against each other, and the micropyramid structure is deformed, thereby increasing the contact area of the ion‐electron pair. Simultaneously, the electric field strength at the contact position increases and a large number of ions in the ion gel is ionized. These ionized ions gather rapidly at the contact interface subject to the action of the electric field force (arrow: direction of ion movement). Furthermore, the ion concentration presents a significant gradient distribution (arrow color), as shown in Figure 2h(ii), which greatly promotes the formation of the EDL effect. To gain further insight into the stress distribution and contact area between the electrode and ionic gel, we also employ COMSOL FEA to investigate the single‐micropyramid and flat‐structure e‐skins, as shown in Figure 2i and Figure S9 (Supporting Information). The flat‐structured e‐skin evenly distributed stress across the planar interface that resulted in large contact areas (high‐initial capacitance); no significant deformation or compression of the structure was observed when subjected to pressure. In contrast, the single‐micropyramid e‐skin features fewer contact points at the interface and generates a highly concentrated stress distribution at the contact points, thus achieving high sensitivity to low pressure. Furthermore, the capacitance at the bottom interface (C_bottom_) of the ionic gel remains almost unchanged due to the complete attachment to the electrode, which is considerably larger than that at the top interface (*C*_top_). Hence, the total capacitance (*C*_total_) is primarily determined by the C_top_. As illustrated in Figure S10 (Supporting Information), C_total_ can be expressed as 1/*C*_total_ = 1/*C*_top_, and *C*_top_ can be equivalent to the numerous *C*_EDL/n_ generated in parallel at the interface between the top electrode and the ionic gel, that is, *C*_top_ = Σ*C*_EDL/n_. From this, it can be concluded that the changes in the contact area between the top electrode and the ionic gel significantly affect the capacitance change of the e‐skin.

The capacitance response of the single‐micropyramid e‐skin in pressure mode is shown in Figure 2j. The sensitivity of the capacitive e‐skin is a key parameter for the evaluation of pressure sensing and is defined as *S* = *δ*(Δ*C*/*C*_0_)/*δP*, where *P* is the applied pressure. The maximum sensitivity of 655.3 kPa^−1^ is exhibited at pressures below 0.5 kPa (an enlarged figure can be found in Figure S11a in the Supporting Information), a linear response corresponds to sensitivity up to 327.9 kPa^−1^ over a pressure range of 0.5–15 kPa (see Note S1 in the Supporting Information for the quantitative relationship between pressure and output signal), and a wide pressure detection range of 150 kPa is observed (Figure S11b, Supporting Information). It is noteworthy that the sensitivity remains unaffected by temperature and humidity, as well as the bending angle of the MAB e‐skin, as shown in Figure S12 (Supporting Information). The flat‐structure e‐skin is also prepared for comparison, and its sensitivity curve is depicted as the blue line in Figure 2j. It is evident that the sensitivity of the single‐micropyramid e‐skin is much higher than that of the flat‐structure, aligning with the expected mechanism outlined above. Additionally, the maximum sensitivity (2.73 kPa^‐1^) for e‐skin with micropyramid structure but with undoped ionic liquid is even lower, as shown in Figure S11c (Supporting Information). The limit of detection (LOD) refers to the minimum pressure that the e‐skin can detect at a baseline pressure of 0 Pa. As shown in Figure 2k, the LOD of this e‐skin is determined to be ≈0.2 Pa, and is superior to human skin (≈100 Pa).^[^ ^36^, ^37^ ^]^ The decrease of Δ*C*/*C*_0_ below the baseline in the curve is caused by the proximity sensing of the approaching object in the vertical direction; when the object moves away, there is no upward trend owing to the removal in the horizontal direction. Simultaneously, we also show the LOD test results based on vertical approach and vertical move away, as shown in Figure S11d (Supporting Information). A comparison between our proposed e‐skin and the recently reported capacitive e‐skin provided in Figure 2l demonstrates excellent performance in terms of sensitivity and LOD performance.^[^ ^38^, ^39^, ^40^, ^41^, ^42^, ^43^, ^44^, ^45^, ^46^, ^47^, ^48^ ^]^ Table S1 (Supporting Information) presents more detailed performance comparisons, including sensitivity, response/recovery time, LOD, and compression stability. Response and recovery times are other essential parameters of the e‐skin, and have been extensively used to evaluate its dynamic response. The introduction of microstructures can accelerate the response and recovery of the e‐skin due to their ability to quickly store and release energy for elastic recovery. We test the response and recovery times of the e‐skin by applying, maintaining, and then removing a pressure of 5 kPa; these are measured as 11.2 and 16.8 ms (Figure 2m), which is far lower than the response time of human skin (30–50 ms).^[^ ^37^, ^49^ ^]^ The fast response and recovery times enable the e‐skin to detect high‐frequency dynamic signals, which are essential for collecting real‐time, continuous, and accurate information in various applications. To evaluate the repeatability of the e‐skin, three loading and unloading cycles are performed at the pressures of 1, 5, and 10 kPa, as shown in Figure S11e (Supporting Information). The capacitance response changes quickly in response to pressure and maintains a stable and repeatable waveform. Mechanical durability is of great importance for the application of e‐skin. We subjected it to repeated compression and release at a pressure of 10 kPa over 10 000 cycles, during which the e‐skin exhibited no significant drift or fluctuation (Figure 2n). As shown in Figure S11f (Supporting Information), taking the signal output of the device at the center of the array that is most affected by bending as an example, the durability test of the array is conducted under 90° bending. It can be observed from the figure that the signal output varies with the bending angle and there is no significant fatigue within 1200 cycles, but the degree of variation is limited. Specifically, despite applying a significant bending angle to the array during testing, the actual amount of bending allocated to this device is relatively restricted due to the small size of the devices in the array (1.3 × 1.3 mm^2^), resulting in a small capacitance response. Overall, the proposed MAB e‐skin exhibits excellent sensing performance in both proximity and pressure modes.

### 2.4 Spatial Mapping in Proximity and Pressure Modes

**Figure 3:**
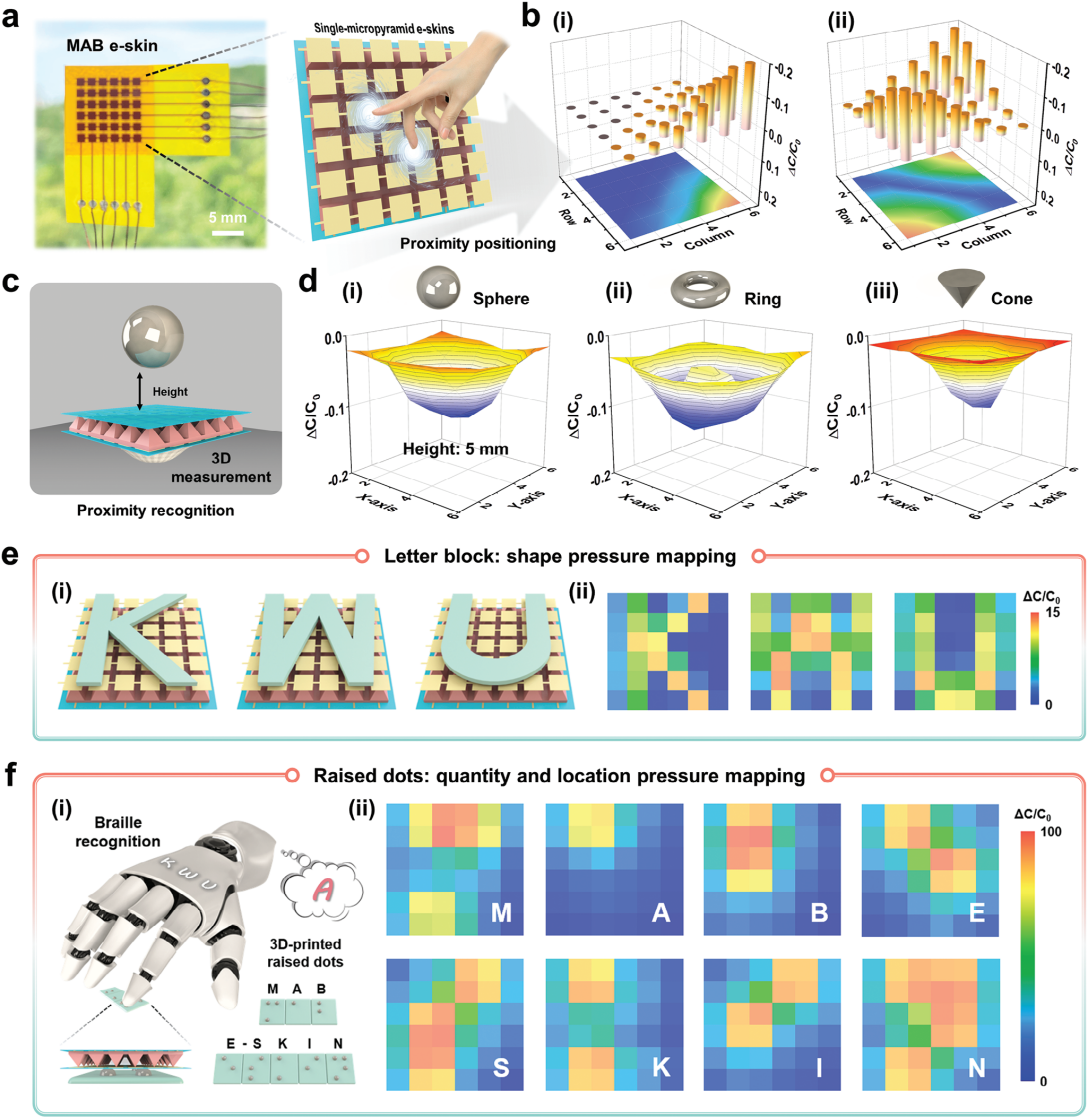
Demonstrations of spatial mappings in proximity and pressure modes. a) Photograph of the MAB e‐skin composed of 36 single‐micropyramid e‐skins. Inset: schematic of proximity positioning of fingertips. b) Spatial proximity mapping corresponding to different positions approached by the fingertips (one i) and two i) fingers). c) Schematic of 3D measurements in proximity mode (take a sphere as an example). d) Detection of various shapes such as sphere i), ring ii), and cone iii). All objectives are 5 mm apart from the center of the MAB e‐skin. e) Schematic of the 3D printed letter blocks on the MAB e‐skin and corresponding shape pressure mapping. f) Robotic index finger with MAB e‐skin touching the raised dots of Braille letter, and corresponding quantity and location pressure mapping.

Superior spatial mapping capability allows robotic skin to perform large‐scale contact and contactless interaction effectively. Fortunately, the MAB e‐skin proposed in this study possesses bimodal sensing capabilities for both proximity and pressure, thus enabling precise positioning and recognition of external stimuli through spatial proximity and pressure mapping information. **Figure** **3**a shows the MAB e‐skin, composed of 36 single‐micropyramid e‐skins, mapping the spatial proximity position of the finger by detecting the capacitance response of every single‐micropyramid e‐skin. As schematically illustrated in Figure 3b(i), when one fingertip approaches the lower right corner of the MAB e‐skin (Figure S13(i), Supporting Information), the channels at the corresponding position generate specific capacitance responses. Figure 3b(ii) demonstrates that the channels of two different positions can simultaneously produce capacitive responses upon approaching two fingertips at the lower left and upper right corners of the MAB e‐skin (Figure S13(ii), Supporting Information). Interestingly, it can be observed that the spatial proximity positions of the fingertips correspond to the capacitance responses (on a one‐by‐one basis), denoting the ability to detect multiple positions concurrently. Furthermore, our MAB e‐skin also accurately recognizes the 3D shapes of objects in a contactless manner. Figure 3c shows the proximity measurement of 3D objects using a sphere as an example. When a metal sphere with a diameter of 10 mm approaches the MAB e‐skin, the capacitance response decreases to −0.032, −0.059, and −0.12 as the height between the sphere and the MAB e‐skin decreases from 25 to 5 mm (Figure S14a,b; Figure 3d(i), Supporting Information). Apart from the proximity height, different object shapes also cause different changes in capacitance responses, thus making it possible to recognize different object shapes. To this end, three different metallic objects, including a sphere, a ring, and a cone, are measured (Figure S15, Supporting Information), where the metal sphere maps a hemispherical 3D shape (Figure 3d(i)); the metal cone has a vertex causing a prominent disturbance in the electric field and resulting in a valley‐like mapping image (Figure 3d(ii)); the mapping image generated by the metal ring clearly shows the inner empty space of the ring (Figure 3d(iii)).

In addition to spatial proximity mapping, spatial mapping in pressure mode is also described and verified as follows. With 3D printed letter blocks shaped like “K”, “W”, and “U” placed on the MAB e‐skin (Figure 3e(i)), the output of the pressure mappings closely matches the contours and weights of the letter blocks (Figure 3e(ii)), thus demonstrating its high‐resolution recognition capability for complex shapes. Braille, a tactile representation of letters and numerical symbols using a basic structure of 6 raised dots; it is a written language specifically designed for the blind that allows reading based on touch. It is also a language used by visually impaired or blind people to communicate and exchange ideas with each other. However, without prior learning of Braille, it is difficult for non‐Braille users to accept and understand this conversational medium. Therefore, Braille translation is important for normal communications between Braille users and non‐Braille users. In view of this, the MAB e‐skin is attached to the index finger of the robot for touch recognition of Braille letters. As shown in Figure 3f(i), the index finger of the robot is controlled to press the 3D printed raised dots (“M”, “A”, “B”, “E”, “S”, “K”, “I”, and “N”) in sequence, the MAB e‐skin generates capacitive response signals in the corresponding single‐micropyramid e‐skins, and the mapped quantity and position corresponds one‐to‐one with the raised dots of Braille letter (Figure 3f(ii)). To better distinguish letter blocks and Braille raised dots, we prepared and assembled a higher‐resolution MAB e‐skin (10 × 10 matrix) (Figure S16a–c, Supporting Information), and the spatial pressure mapping corresponding to letter blocks and Braille raised dots is shown in Figure S16d–f (Supporting Information). As can be observed from the figure, the shapes of different letter blocks can be displayed more clearly; the raised dots of different Braille letters can be distinguished more effectively. Undoubtedly, the proposed MAB e‐skin demonstrates remarkable capabilities for spatial bimodal mapping in proximity and pressure modes, thus offering new research ideas for endowing robotic skin with multifunctional and large‐scale sensing capabilities.

### 2.5 Intelligent Material Perception in Proximity Mode

**Figure 4:**
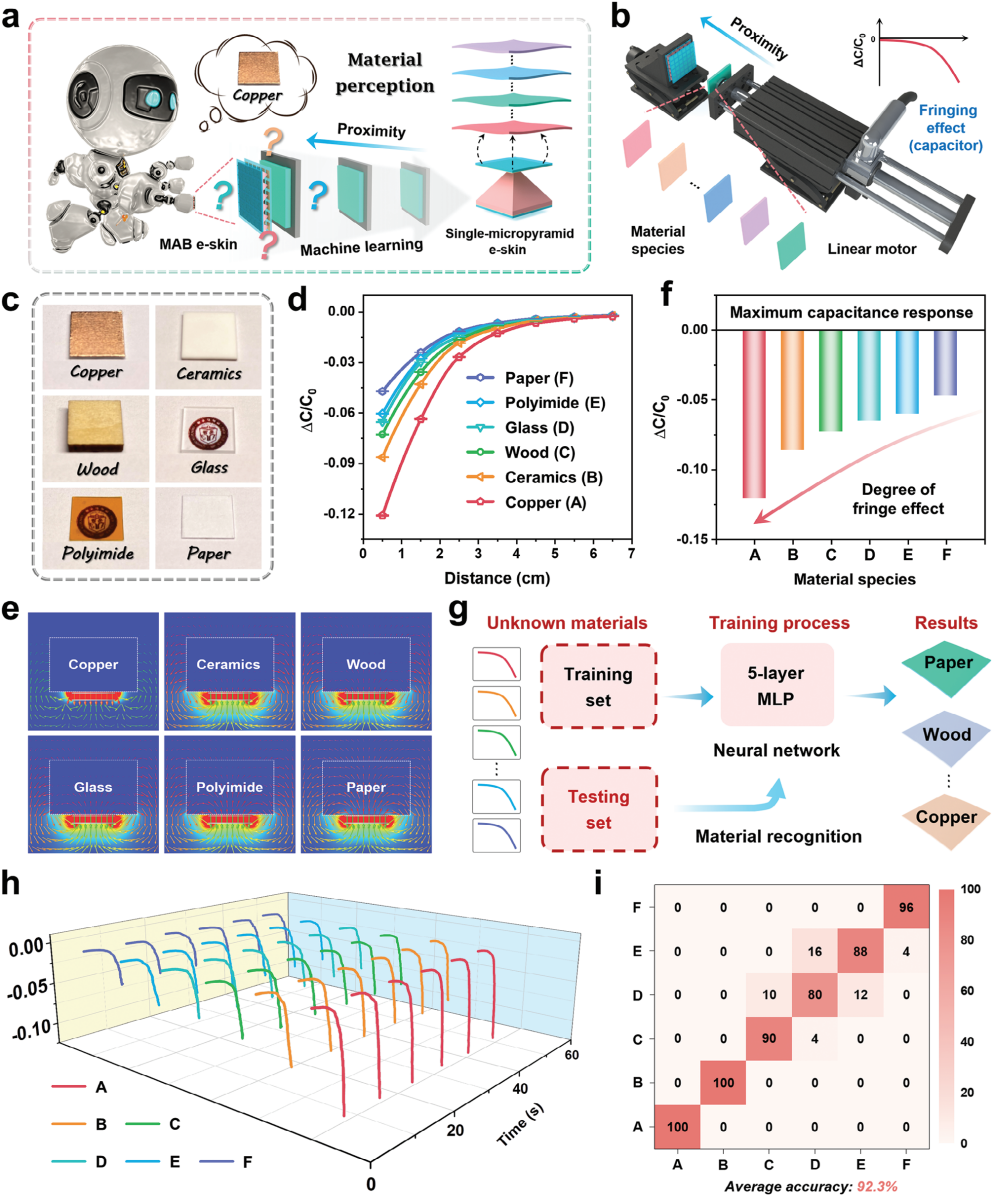
Demonstrations of intelligent material perception in proximity mode. a) Concept diagram of the designed intelligent material perception. b) Schematic of the intelligent material perception test system. c) Photographs of 6 tested materials. d) Capacitance response of 6 materials at various distances from the MAB e‐skin. e) COMSOL simulation results for the electric field line distribution of different materials near the e‐skin. f) Capacitance response of 6 materials with the MAB e‐skin at 0.5 cm. g) MLP‐motivated material perception process flow, including training and recognition. h) Capacitance response waveforms generated by 6 materials in proximity mode. i) Confusion matrix showing the classification accuracy (%) for the material perception.

The human brain is capable of helping our skin perceive accurately the shape, size, and texture of different objects based on long‐term learning and training. However, the skin is unable to perceive the material properties of objects featuring flat surfaces with indistinguishable characteristics. By combining the proposed MAB e‐skin with the MLP neural network, it is possible to perceive the material properties of the object as it gradually approaches the MAB e‐skin, thus exhibiting tactile perception abilities beyond those of human beings (**Figure** **4**a). Therefore, we have established an intelligent material perception test system in proximity mode (Figure 4b) and selected 6 materials with flat and indistinguishable surfaces as representative test materials. The MAB e‐skin and the tested material are installed on fixed and movable platforms, respectively, on which the linear motor can precisely control the moving speed. As different materials approach the MAB e‐skin, the single‐micropyramid e‐skin located at the central position of the array (area: 1.3 × 1.3 mm^2^) generates significantly different capacitance response curves. The reason underlying the aforementioned situation is mainly attributed to the coupling of the fringing and the mutual capacitance effects, which are influenced by the inherent dielectric constant of the tested material. Notably, since the area of the tested objects far exceeds 1.3 × 1.3 mm^2^, the effective corresponding area is certain regardless of the shape of the objects, which effectively excludes the influence of different shapes of objects on the accuracy of material perception, so that the change in capacitance response is mainly related to the material of the object. As a proof of concept, capacitance response tests are performed on 6 materials (Figure 4c), including copper, ceramics, wood, glass, polyimide, and paper are performed, during the period MAB e‐skin is approached. To observe the capacitance responses at uniform intervals, we extract 7 points within the range of 0.5‐6.5 cm (1 cm intervals) and fit the capacitance response curves, as shown in Figure 4d and Figure S17 (Supporting Information). It can be observed that the capacitance response curve varies as a function of the dielectric constant in different materials. Notably, materials with a larger dielectric constant, such as metals (copper), yield a more pronounced capacitance responses. Figure 4e shows the COMSOL simulations on the electric field line distribution of the aforementioned 6 materials, whereby copper (with a larger dielectric constant) exhibits an obvious shunt phenomenon to the electric field lines. As the dielectric constant of different materials decreases, the shunt phenomenon gradually disappears, which is consistent with the proximity sensing mechanism, that is, the more pronounced the shunt phenomenon, the larger the capacitance response. Figure 4f and Figure S18 (Supporting Information) show the maximum capacitance response and sensitivity of 6 materials, respectively, which more intuitively display differences in their capacitance response curves, thus further indicating the possibility for the MAB e‐skin in proximity mode to infer the material species. Relying on the above discovery, the intelligent perception of different materials is successfully achieved by incorporating a 5‐layer MLP neural network. The material perception process is presented in Figure 4g and Figure S19 (Supporting Information). First, we utilize the MLP neural network to construct the training model (see Note S2 for the detailed model construction process) with an input layer, three hidden layers, and an output layer (Figure S20, Supporting Information). Next, the capacitance response curves of 6 materials, which are collected in the range of 0.5–10.5 cm at a speed of 15 mm·s^‐1^, are selected as the datasets. During the training and optimization of the MLP neural network, 80% of the datasets of each material are randomly selected as the training samples, and the remaining 20% are used as test samples to validate the trained 5‐layer MLP neural network. During this process, the extracted capacitance response curves of the 6 materials are presented in Figure 4h. It can be observed that there are significant differences in the capacitance response curves between different materials, whereas the multiple curves collected from the same material are highly consistent. Notably, the samples in the datasets are diverse, including data collected at multiple time points (differences in temperature and humidity) to avoid the impact of environmental factors on the accuracy of material perception. Figure 4i presents the confusion matrix (each row represents a test sample in an actual class and each column represents a predicted class) of the ML results used to predict 6 materials, with an average prediction accuracy of 92.3%. These results demonstrate the excellent classification ability of the constructed MLP neural network in terms of the perceptions of material species in proximity mode.

### 2.6 Intelligent Surface Shape Perception in Pressure Mode

**Figure 5:**
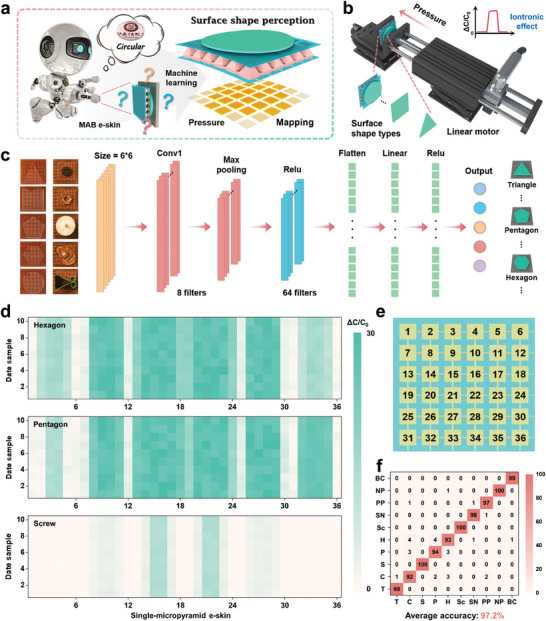
Demonstrations of intelligent surface shape perception in pressure mode. a) Concept diagram of the designed intelligent surface shape perception. b) Schematic of the intelligent surface shape perception test system. c) Schematics of the process and parameters for constructing the CNN model. d) Distribution diagram of 10 sets of capacitance response‐pressure mapping data collected by the circular, pentagon, and screw. e) 36 single‐micropyramid e‐skins with corresponding numbers. f) Confusion matrix showing the classification accuracy (%) for surface shape perception.

In practical applications, the precise perception of an object often requires the combined action of multiple perception elements. Although the MAB e‐skin has achieved material perception in proximity mode, this alone is not sufficient. We also need to cooperate by perceiving the surface shape of the object in pressure mode. As shown in Figure 3e,f, the MAB e‐skin exhibits high‐resolution spatial pressure mapping capability, thus indicating the possibility of perceiving the surface shape of an object in pressure mode. Based on this discovery, we integrate the MAB e‐skin with a CNN to enable the perception of surface shapes in the forms of capacitance response‐pressure mappings (**Figure** **5**a). Specifically, using a test system similar to the one described earlier for material perception, 5 glass plates with different surface shapes (triangle (bottom edge: 1 cm), circular (diameter: 1 cm), square (edge: 1 cm), pentagon (diagonal: 1 cm), and hexagon (diagonal: 1 cm)) (Figure S21a, Supporting Information) and 5 common objects (screw, screw nut, push pin, nose pad, and binder clip) (Figure S21b, Supporting Information) are sequentially mounted on the movable platform of a linear motor. Subsequently, the capacitance response‐pressure mapping generated by 36 single‐micropyramid e‐skins is investigated at constant pressure, as shown in Figure 5b and Figure S22 (Supporting Information). Further, we also employ a 10 × 10 matrix‐based MAB e‐skin to characterize the capacitance response‐pressure mapping of 5 common objects, aiming to enhance their fidelity at a higher resolution, as illustrated in Figure S23 (Supporting Information). To investigate the impact of bending on capacitance response‐pressure mapping, we placed different surface shapes on MAB e‐skin with bending angles of 30° and 60°, and the results are shown in Figure S24a (Supporting Information). Owing to the e‐skin (6 × 6 matrix) being in a curved state, it is difficult for these 5 surface shapes to fully fit, resulting in the pressure mainly concentrated on the middle two rows of sensing units. Despite the bending deformation of the e‐skin has an impact on capacitance response‐pressure mapping, significant differences in 5 surface shapes can still be observed. Meanwhile, we further apply the 10 × 10 matrix‐based MAB e‐skin for characterizing the capacitance response‐pressure mapping under bending deformation, as shown in Figure S24b (Supporting Information). It can be observed that a higher resolution array demonstrates more pronounced differences in 5 surface shapes. A CNN model consisting of the convolution layer, pooling layer, flatten layer (2D to 1D), and fully connected layer is then constructed for feature extraction and automatic perception of capacitance response‐pressure mapping using different surface shapes (Figure 5c). The signal from each single‐micropyramid e‐skin is recorded with 1 data point (36 single‐micropyramid e‐skins), and 100 samples are collected for each surface shape. To strengthen the dataset, the 100 collected samples are noise‐augmented to create a new dataset with 1000 samples. Of these, 900 samples (90%) are used to train the CNN model, while the remaining 100 samples (10%) are used to test its accuracy. In this process, 10 sets of capacitance response‐pressure mapping data for triangle, circular, square, pentagon, hexagon, screw, screw nut, push pin, nose pad, and binder clip are shown in Figure 5d and Figure S25 (Supporting Information), where the abscissa represents 36 single‐micropyramid e‐skins of the MAB e‐skin (their numbering is the same as that in Figure 5e). Importantly, the capacitance responses generated by each single‐micropyramid e‐skin are basically consistent, thus indicating that the MAB e‐skin is highly stable in multi‐channel data acquisitions and provides the necessary guarantee for accurate perception of surface shapes. Figure 5f shows the confusion matrix of the classification results for 10 surface shapes, with each row representing the actual surface shapes and each column representing the predicted surface shape. The results show that the average accuracy for the 10 surface shapes is 97.2%. The reason why this example only demonstrates simple shapes is because of two aspects: first, the current limitations of micro‐processing technologies make it difficult to prepare e‐skins cost‐efficiently with higher resolution in an area of 1 × 1 cm^2^; second, the current limitations of acquisition technologies make it difficult to add more acquisition channels due to the time and cost involved. In the future, with the continuous improvement of micro‐processing and acquisition technologies, it is possible to perceive surface shapes with more complex and smaller differences. In a word, the proposed ML‐motivated MAB e‐skin successfully achieves the accurate perception of different materials and surface shapes within one proximity‐pressure cycle; this is anticipated to provide new tactile perception abilities for bionic robots and will promote the development of intelligent perception.

## 3 Conclusion

By combining the fringing and the iontronic effects, we develop a high‐performance MAB e‐skin based on capacitive sensing and demonstrate its potential applications in intelligent perception. The micropyramid array of the ionic gel is prepared by adopting cost‐effective and highly efficient light‐curing 3D printing technology. The MAB e‐skin in pressure mode displays a range of excellent sensing performances, including high sensitivity to tiny pressures, linear sensitivity over a wide detection range, low LOD, fast response/recovery time, and good durability, which are all indispensable for the development of practical intelligent perception. Leveraging its bimodal sensing characteristics, the MAB e‐skin achieves contactless measurements of 3D objects and contact recognition of Braille letters, thus indicating its superior spatial mapping capabilities in both proximity and pressure modes. As the highlight of this work, we further demonstrate the intelligent material and surface shape perception testing system by applying MLP and CNN models to train and learn the response curve in the proximity mode and 36‐channel response‐pressure mapping in pressure mode collected by the MAB e‐skin. The testing system can sequentially perceive 6 different materials and 10 surface shapes within one proximity‐pressure cycle, with respective average accuracies of 92.3% and 97.2%. While capacitive sensing‐based intelligent perception is still in its infancy, it has shown tremendous potential in material and surface shape perception and has already surpassed human tactile perception. Going forward, the proposed MAB e‐skin design structure, bimodal sensing mechanism, and intelligent perception architecture are anticipated to introduce new ideas for the development of truly intelligent robotic skin or blind perception and will inspire AI‐based cutting‐edge applications.

## 4 Experimental Section

### Preparation of Anti‐Micropyramid Array Template

An anti‐micropyramid array (6 × 6 matrix) template was designed and sliced (slice thickness of 50 µm) using 3D modeling software (AutoCAD), and then fabricated by curing commercially available photosensitive resin (RSN526) layer‐by‐layer using a digital light processing 3D printer (Octavelight R1‐30 µm, Dongguan Baduguang Technology Co., Ltd, China). After accomplishing the 3D printing process, the printed anti‐micropyramid array template was carefully separated from the constructed platform, rinsed with ethanol for 5 min to remove the residual uncured photosensitive resin, and post‐cured in a UV LED (wavelength: 405 nm) for 30 min to enhance the mechanical properties of the printed anti‐micropyramid array template (Figure 1c(i)).

### Preparation of PVDF‐HFP/[EMIM][TFSI] Ionic Gel with Micropyramid Array

Initially, 2 g of PVDF‐HFP (Macklin, China) was dissolved in 18 g of acetone and stirred at room temperature until it completely dissolved to form a transparent gel. [EMIM][TFSI] (Aladdin, China) with a weight ratio of 50% was then added dropwise and stirred for 30 min. To prepare the micropyramid array, PVDF‐HFP/[EMIM][TFSI] ionic gel was poured onto the anti‐micropyramid array template, and the bubbles were removed in a vacuum drying oven to fill the solution fully. After natural drying, it was easy to peel off and obtain a PVDF‐HFP/[EMIM][TFSI] ionic gel with the micropyramid array (Figure 1c(ii)).

### Assembly of MAB E‐Skin

The MAB e‐skin was assembled based on a typical “sandwich” structure, namely PI/Cu/Au electrode array, PVDF‐HFP/[EMIM][TFSI] ionic gel with micropyramid array, and Au/Cu/PI electrode array (Figure 1c(iii)). Among them, the Au/Cu/PI electrode arrays (total area: 1.4 × 3.5 cm^2^, unit area: 1.3 × 1.3 mm^2^) were designed (Figure S4a,b, Supporting Information) using the Jialichuang EDA and were commercially fabricated (Figure S4c, Supporting Information), and the copper wires were welded to the electrode array through solders. During assembly, the two electrode arrays (6 × 6 matrix) were aligned perpendicular to each other, and 36 single‐micropyramid structures were arranged evenly at the intersection of the electrode arrays. The alignment was achieved manually by using an optical microscope.

### Characterization and Measurements

The microstructure morphologies were characterized through SEM (Regulus‐8100, Hitachi). The capacitance measurements were recorded on an Agilent (E4980A) Precision LCR meter. The test system in proximity and pressure modes was driven by a linear motor (PS01, LinMot, Switzerland).

### COMSOL FEA of the EDL Effect

The EDL effect in the MAB e‐skin was simulated by COMSOL FEA, where the EDL was modeled using the Gouy‐Chapman‐Stern model. According to the Gouy‐Chapman theory,^[^ ^50^ ^]^ the diffuse double layer was regarded as a multiphysics coupling of the Nernst‐Planck equation for mass transport of all of the ions, with Poisson's equation (Gauss's law) for the charge density and electric field. The combination of these equations was often referred to as the Poisson‐Nernst‐Planck equation or Nernst‐Planck‐Poisson equation. The Stern correction to the Gouy‐Chapman theory took into account the physical limitations of ion size, considering ions to have a finite size and thus not to be too close to the electrode surface, but at a certain distance from it. By combining the “Electrostatics” and “Transport of diluted species” modules, the concentration gradient distribution of ions in ion gels was calculated and displayed. The fluxes (*J_i_*, SI unit: mol/(m^2^ s)) of the ions were described by the Nernst‐Planck equation:

(1) $$J_{i}=-D_{i}\nabla c_{i}-u_{m,i}z_{i}{\mathrm{Fc}}_{i}\nabla \Phi$$

with *D_i_* (SI unit: m^2^/s) being the diffusion coefficient, *u_m,i_* (SI unit: s·mol/kg) the mobility, *F* (SI unit: C/mol) the Faraday constant, and Φ (SI unit: V) the electric potential in the electrolyte phase.

## Conflict of Interest

The authors declare no conflict of interest.

## Author Contributions

H.N. and X.W. contributed equally to this work. H.N. and Y.L. conceived this work. H.N. and X.W. designed, prepared, and tested the MAB e‐skin. H.N., H.L., and X.W. developed the overall intelligent gesture perception system and verified the feasibility of this architecture. F.Y., W.W., and R.S. helped with the experiments design and intelligent gesture perception system preparation. H.N., E.K., Y.S., and Y.L. participated in the discussion of experimental results. H.N., X.W., and Y.L. wrote the manuscript. N.K. and Y.L. supervised the project. All authors reviewed and commented on the manuscript.

## Supporting information

Supporting Information

## Data Availability

The data that support the findings of this study are available in the supplementary material of this article.
