# Supplementary material for: Micropyramid Array Bimodal Electronic Skin for Intelligent Material and Surface Shape Perception Based on Capacitive Sensing
Source: Adv Sci (Weinh). 2023 Nov 29;11(3):2305528. doi: 10.1002/advs.202305528 (PMC10797442; doi:10.1002/advs.202305528)
Supplement: Supplementary file 1 — Supporting Information [file ADVS-11-2305528-s001.pdf]

ADVANCED SCIENCE
Open Access

Supporting Information

for *Adv. Sci.*, DOI 10.1002/advs.202305528

Micropyramid Array Bimodal Electronic Skin for Intelligent Material and Surface Shape Perception Based on Capacitive Sensing

Hongsen Niu, Xiao Wei, Hao Li, Feifei Yin, Wenxiao Wang, Ryun-Sang Seong, Young Kee Shin, Zhao Yao, Yang Li, Eun-Seong Kim* and Nam-Young Kim**

Supporting Information

Micropyramid Array Bimodal Electronic Skin for Intelligent Material and Surface Shape Perception Based on Capacitive Sensing

Hongsen Niu, Xiao Wei, Hao Li, Feifei Yin, Wenxiao Wang, Ryun-Sang Seong, Young Kee Shin, Zhao Yao, Yang Li, Eun-Seong Kim,* Nam-Young Kim**

H. Niu, F. Yin, W. Wang, R.-S. Seong, E.-S. Kim, Prof. N.-Y. Kim
RFIC Centre
Department of Electronics Engineering
NDAC Centre
Kwangwoon University
Seoul, 01897, South Korea
E-mail: nykim@kw.ac.kr

Prof. Y. Li
School of Microelectronics
Shandong University
Jinan, 250101, China
E-mail: yang.li@sdu.edu.cn

X. Wei, H. Li
School of Information Science and Engineering
University of Jinan
Jinan, 250022, China

Prof. Z. Yao
College of Micro & Nano Technology
Qingdao University
Qingdao, 266071, China

Prof. Young Kee Shin, Prof. N.-Y. Kim
Department of Molecular Medicine and Biopharmaceutical Sciences
Seoul National University
Seoul, 08826, South Korea

Supplementary Note

Note S1. Quantitative relationship between pressure and output signal.

According to the trend of the curves, sensitivity curves within two pressure ranges, 0-0.5 kPa and 5-15 kPa, are separately linearly fitted using Origin software and defined as two linear functions: y=655.3x-1.2 (0-0.5 kPa); y=327.9x+336.5 (5-15 kPa). With these two linear functions, the quantitative relationship between pressure and the output signal can be clearly clarified, and pressure can be quantitatively reversed from the output signal.

Note S2. Construction of MLP neural network model for intelligent material perception.

MLP is constructed by the Keras open-source artificial neural network library, which has five layers, including one input layer, three hidden layers, and one output layer. The 46 neurons in the input layer represent the 46 data points collected by objects of different materials in the process of approaching the MAB e-skin, and the 6 neurons in the output layer represent the output recognition probability of the 6 materials. Regarding the hidden layers, the number of neurons in each layer is set to 64, 128, and 64, respectively (Figure 3d). The input and hidden layers in the 5-layer MLP network use the ReLU activation function to reduce linearization, and the output layer performed the probabilistic output using the SoftMax activation function, as shown in Figure S12.

Supplementary Figure

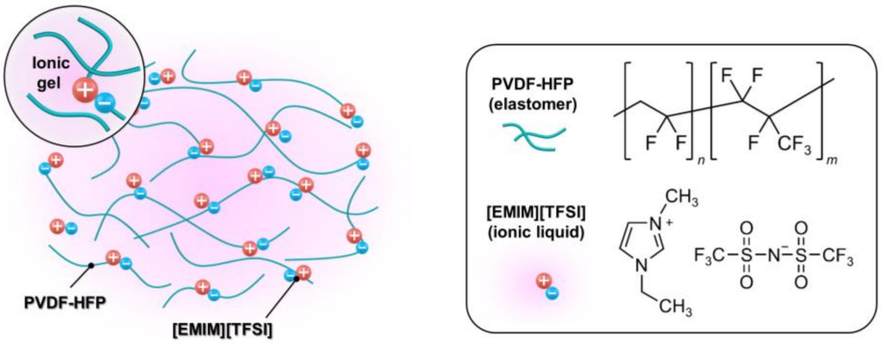

Figure S1. Chemical structures of the two components used in the ionic gel: PVDF-HFP (elastomer) and [EMIM][TFSI] (ionic liquid).

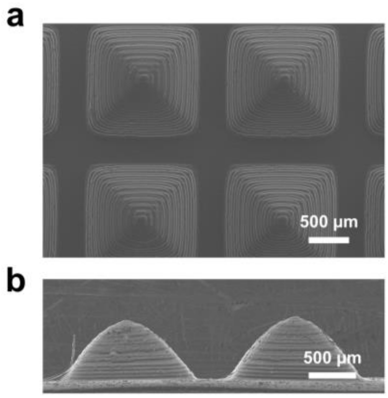

Figure S2. a) Top-view and b) cross-sectional SEM images of the ionic gel with micropyramid array.

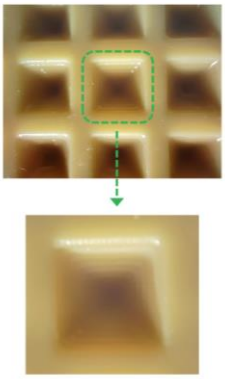

Figure S3. Enlarged image of the anti-micropyramid array template.

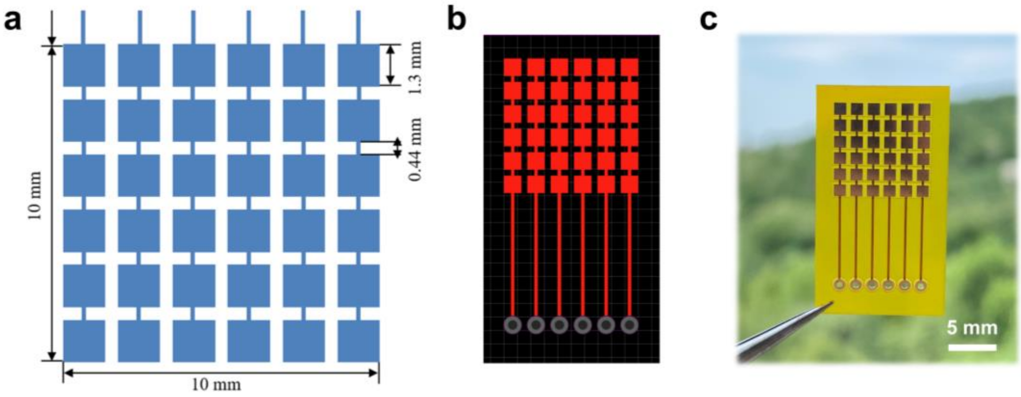

Figure S4. a,b) Schematic and c) photograph of the PI/Cu/Au electrode array.

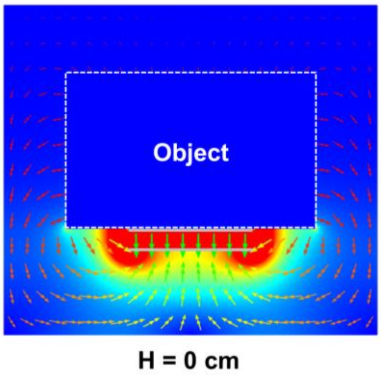

Figure S5. COMSOL simulation for the potential distribution of an object near the single-micropyramid e-skin at H = 0 cm.

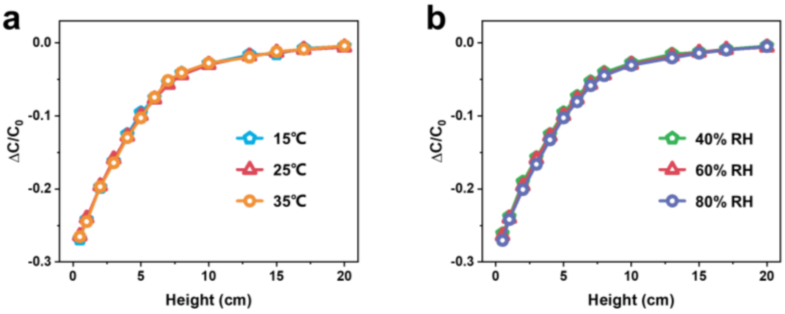

Figure S6. Relative capacitance variations in response to an approaching palm at different a) temperatures and b) humidities.

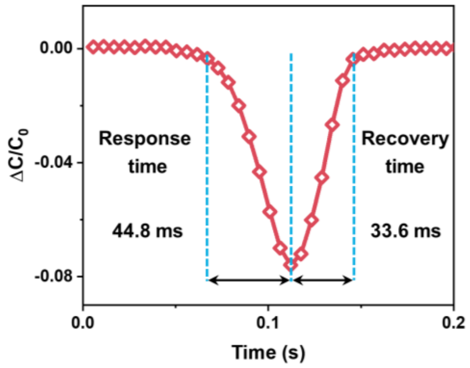

Figure S7. Response/recovery time curve of proximity sensing in the high-frequency movement cycle of Figure 2f.

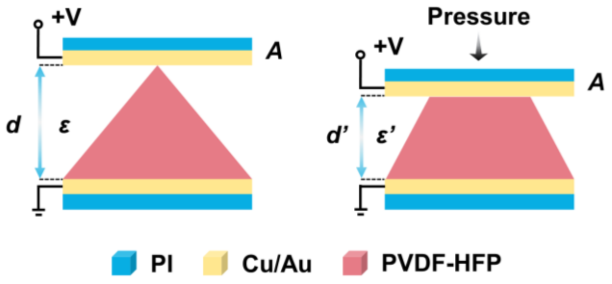

Figure S8. Sensing principles of the conventional parallel-plate capacitive e-skin.

Conventional parallel-plate capacitive sensing:

For the parallel-plate capacitive e-skin, its capacitance (C) can be expressed as

$$C = \varepsilon A/4k\pi d$$

where ε represents the effective dielectric constant; A is the effective area of parallel plates; k stands for electrostatic constant; d means the separation distance between parallel plates. According to the above formula, ε, A, and d can all cause changes in capacitance. However, as the e-skin is not stretchable (A remains constant), the capacitance will be primarily determined by the ε and d, as illustrated in Figure S6. Further, following the general Lichterecker mixing rule,[1,2] the expression for ε between parallel plates is

$$\varepsilon^{\alpha} = V_{\mathrm{air}}\varepsilon_{\mathrm{air}}^{\alpha} + V_{\mathrm{P}}\varepsilon_{\mathrm{P}}^{\alpha}$$

where α is the parameter determining the type of mixing rule; $\varepsilon_{\mathrm{air}}$ and ε_{P} are the dielectric

constant of the air and PVDF-HFP, respectively; V_{air} and V_P are the volume fractions ($V_{air} + V_P = 1$) of air and PVDF-HFP, respectively. As the dielectric between parallel plates consists of air with low ε_{air} (= 1) and PVDF-HFP with high ε_P (~ 9), a large amount of air is squeezed out upon applied pressure, thus leading to an enhanced ε^{α}.

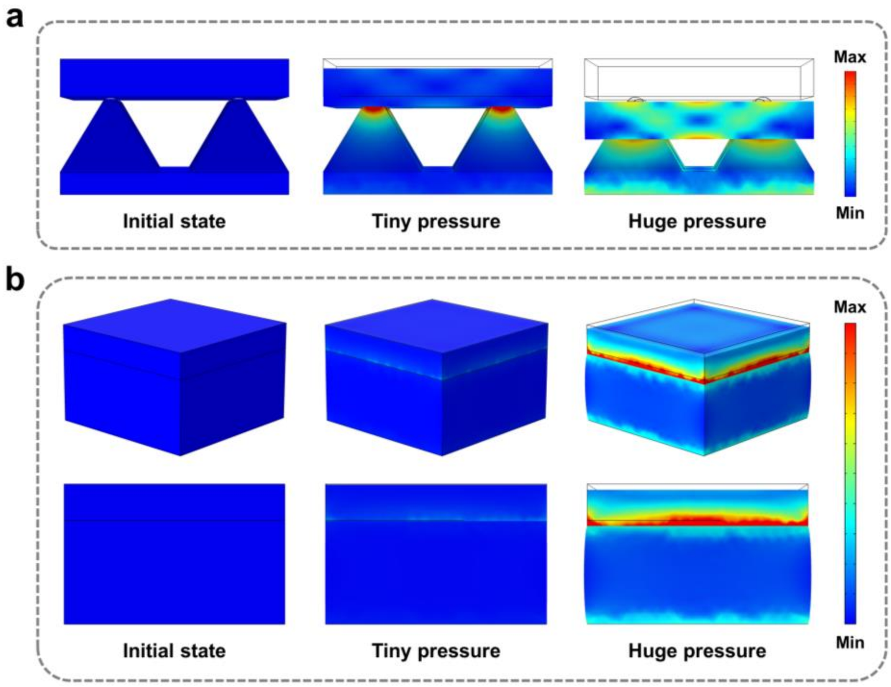

Figure S9. a) Stress distributions of COMSOL FEA simulation results for the single-micropyramid e-skin at various pressures. b) Stress distributions of COMSOL FEA simulation results for the flat-structure e-skin at various pressures.

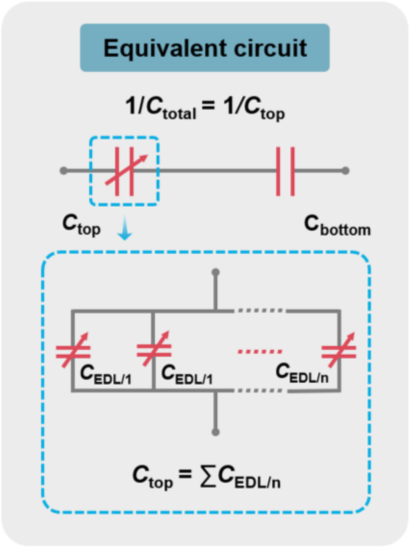

Figure S10. Equivalent circuit of the MAB e-skin in pressure mode.

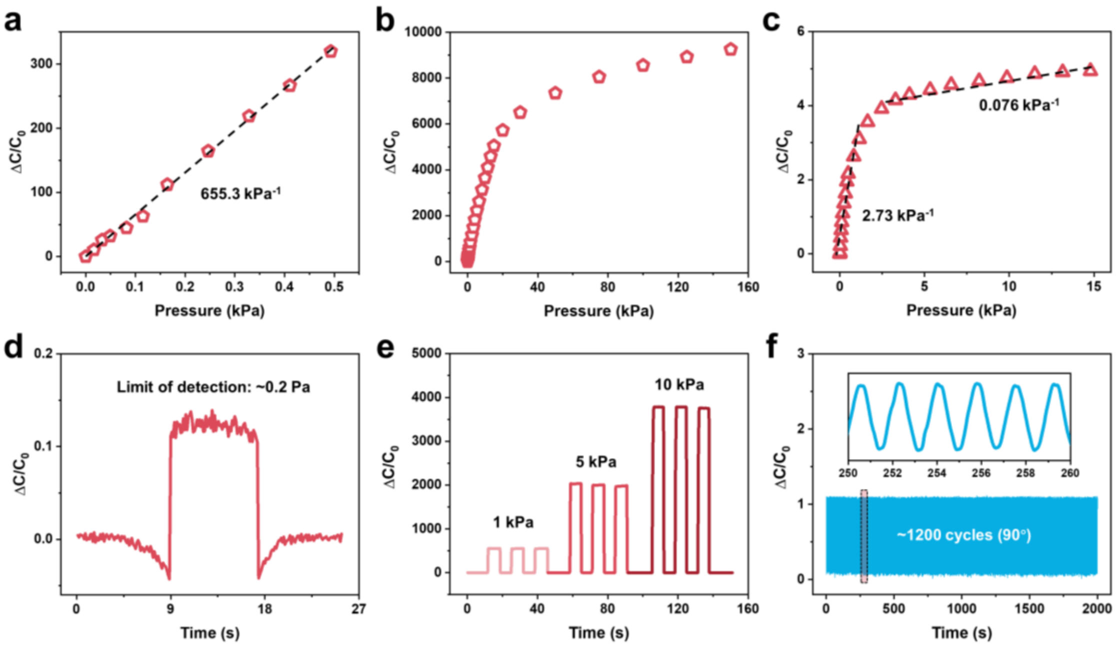

Figure S11. a) Pressure-dependent (<0.5 kPa) relative capacitance changes in the single-micropyramid e-skin. b) Pressure-dependent (150 kPa) relative capacitance changes in the single-micropyramid e-skin. c) Pressure-dependent relative capacitance changes in the single micropyramidal e-skin but without ionic liquid doping. d) LOD of ~0.2 Pa (vertically approaching and moving away). e) Repeatability test at the pressures of 1 kPa, 5 kPa, and 10 kPa. f) Stability tested over 1200 cycles at a bending angle of 90°.

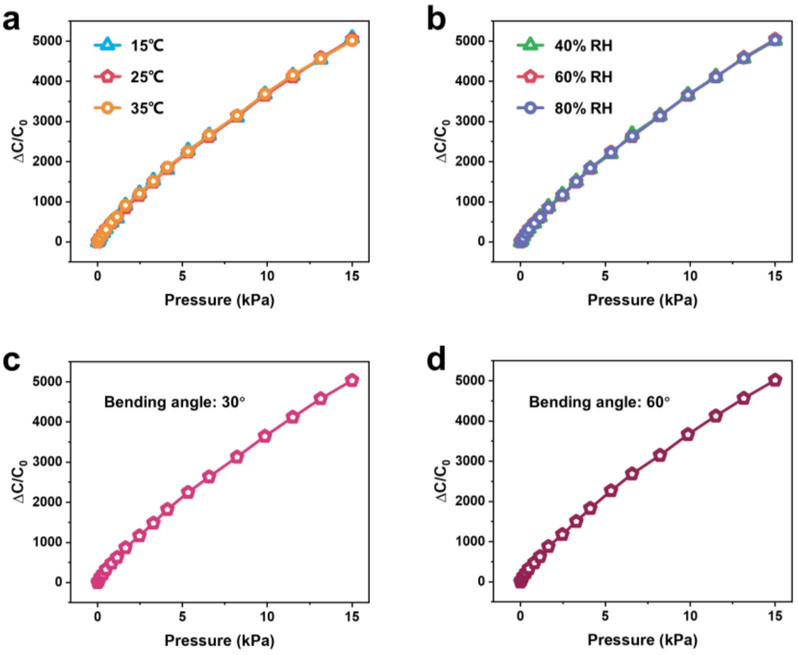

Figure S12. Pressure-dependent relative capacitance changes at different a) temperatures and b) humidities. Pressure sensitivity at bending angles of c) 30° and d) 60°.

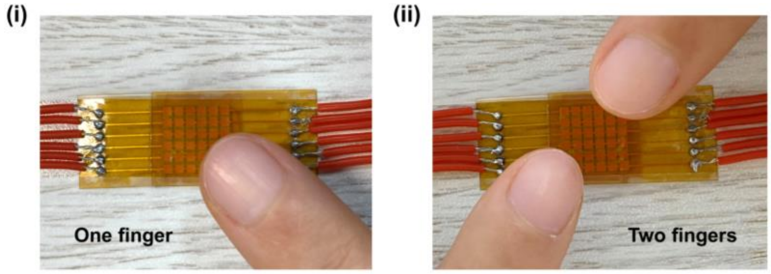

Figure S13. Photographs of different positions approached by the fingertips (one (i) and two (i) fingers).

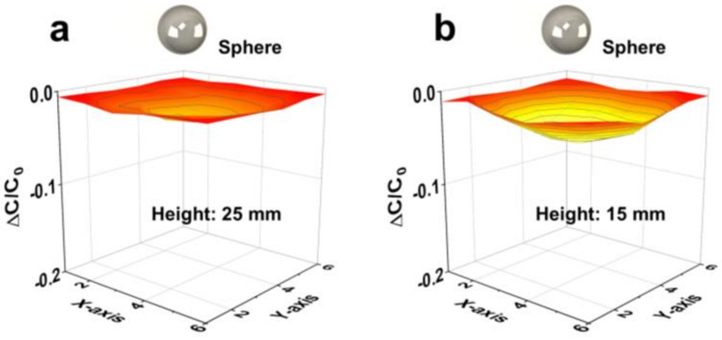

Figure S14. 3D mappings of capacitive responses at two different heights between the object

and MAB e-skin.

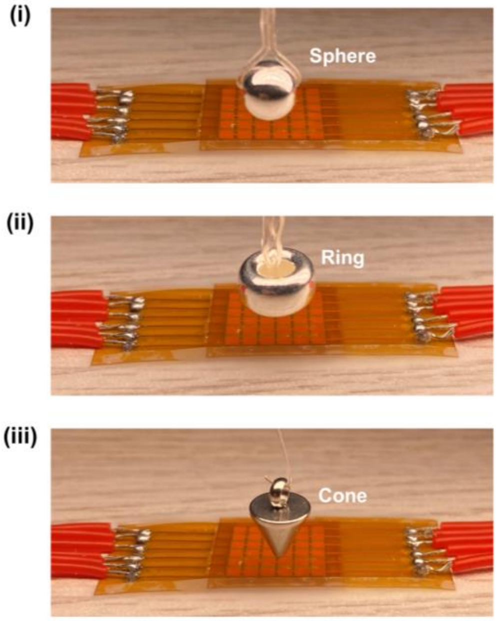

Figure S15. Photograph of 3D measurement and metallic objects such as sphere (i), ring (ii), and cone (iii). All objects are 5 mm apart from the MAB e-skin.

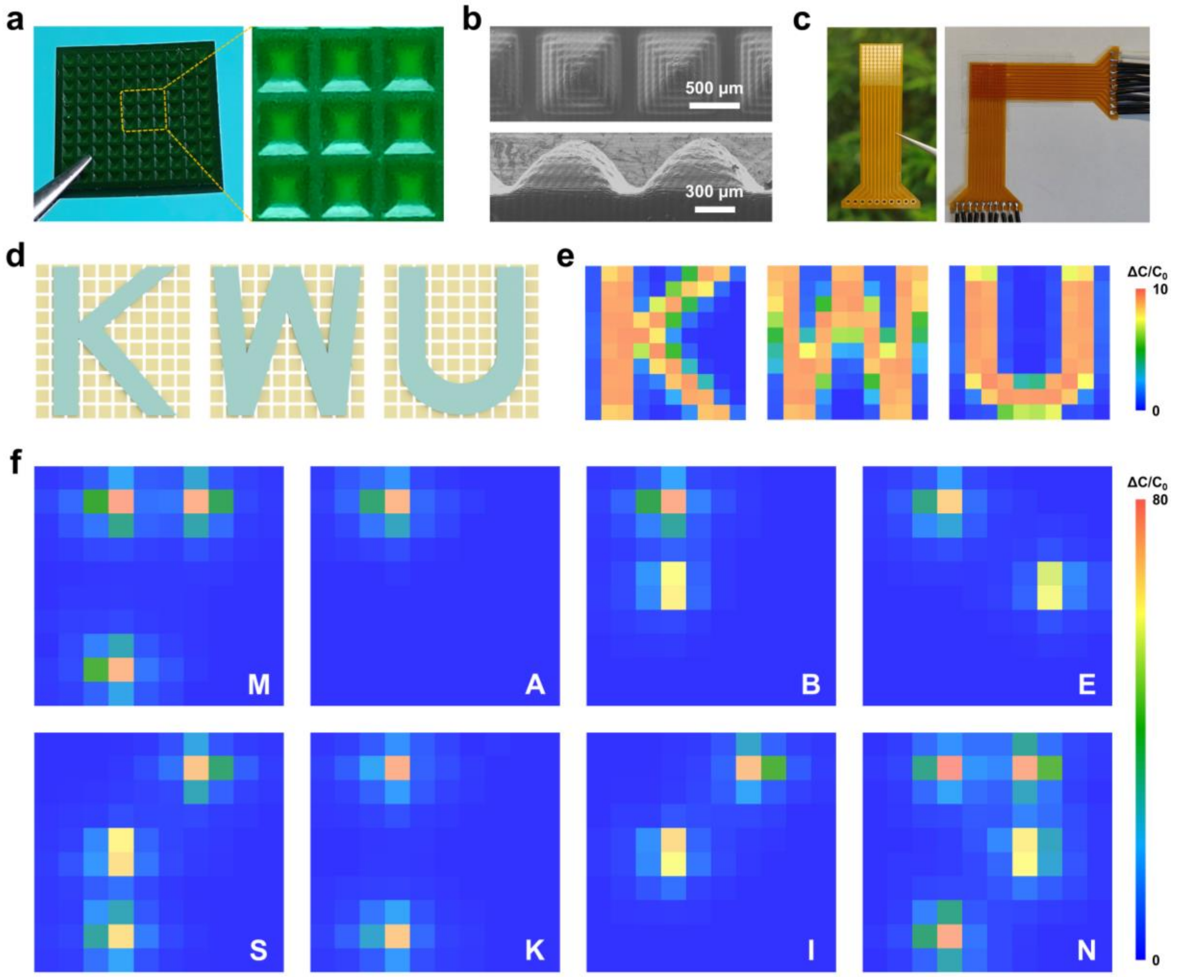

Figure S16. a) Photograph of the anti-micropyramid array template. b) SEM images (top-view and cross-sectional) of ionic gel with micropyramid array. c) Photograph of the PI/Cu/Au electrode array and the MAB e-skin (10 × 10 matrix). d,e) Schematic of the 3D printed letter blocks on the MAB e-skin and corresponding shape pressure mapping. f) Quantity and location pressure mapping of the raised dots of Braille letter.

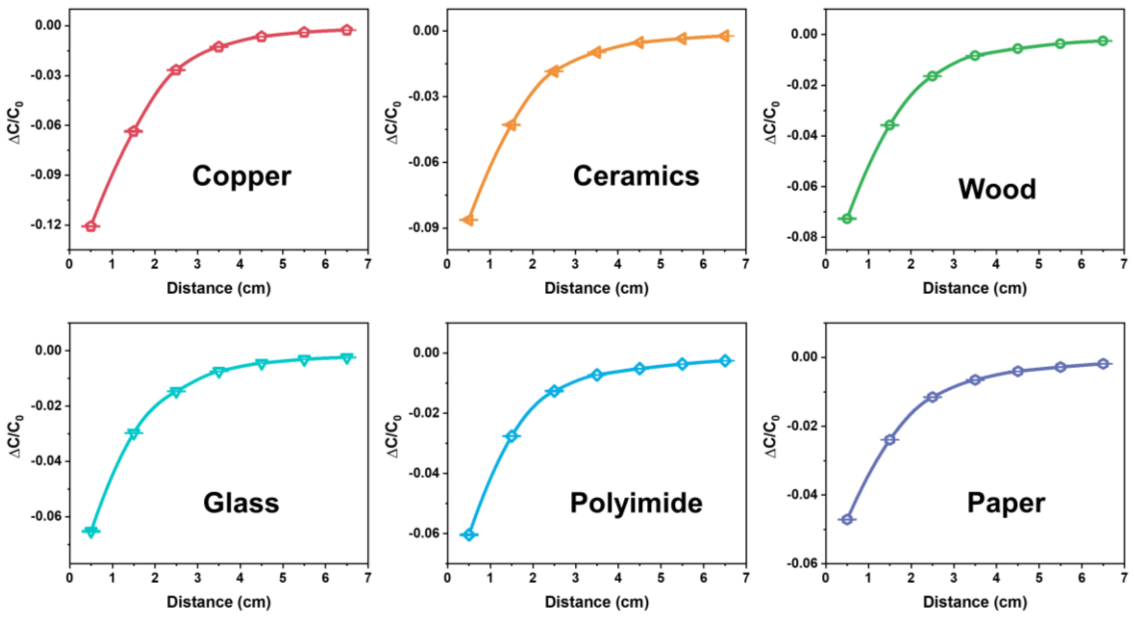

Figure S17. Capacitance response of 6 materials at various distances from the MAB e-skin.

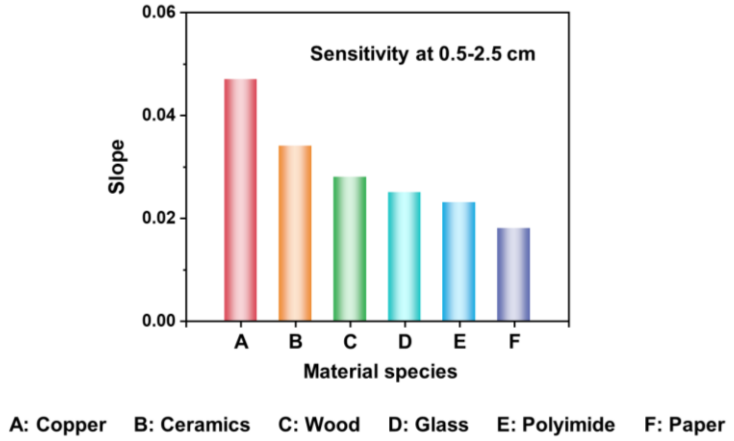

Figure S18. Sensitivities of 6 materials with the MAB e-skin in the distance range of 0.5-2.5 cm.

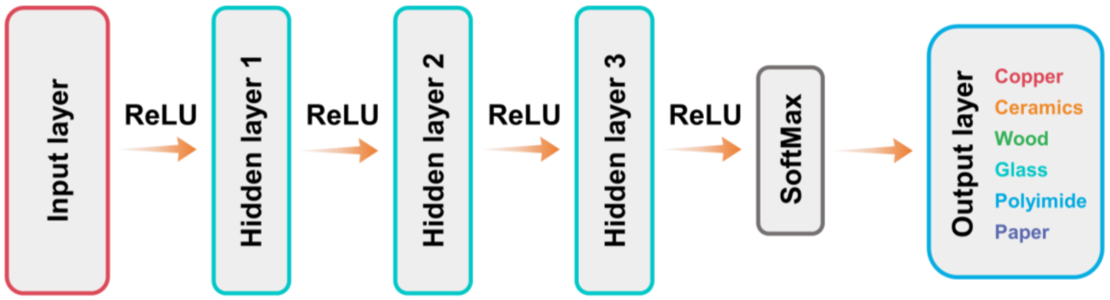

Figure S19. Schematic of the 5-layer MLP neural network model.

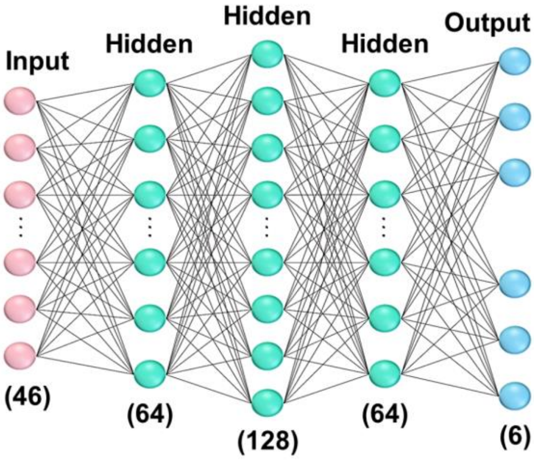

Figure S20. Structure of the 5-layer MLP model.

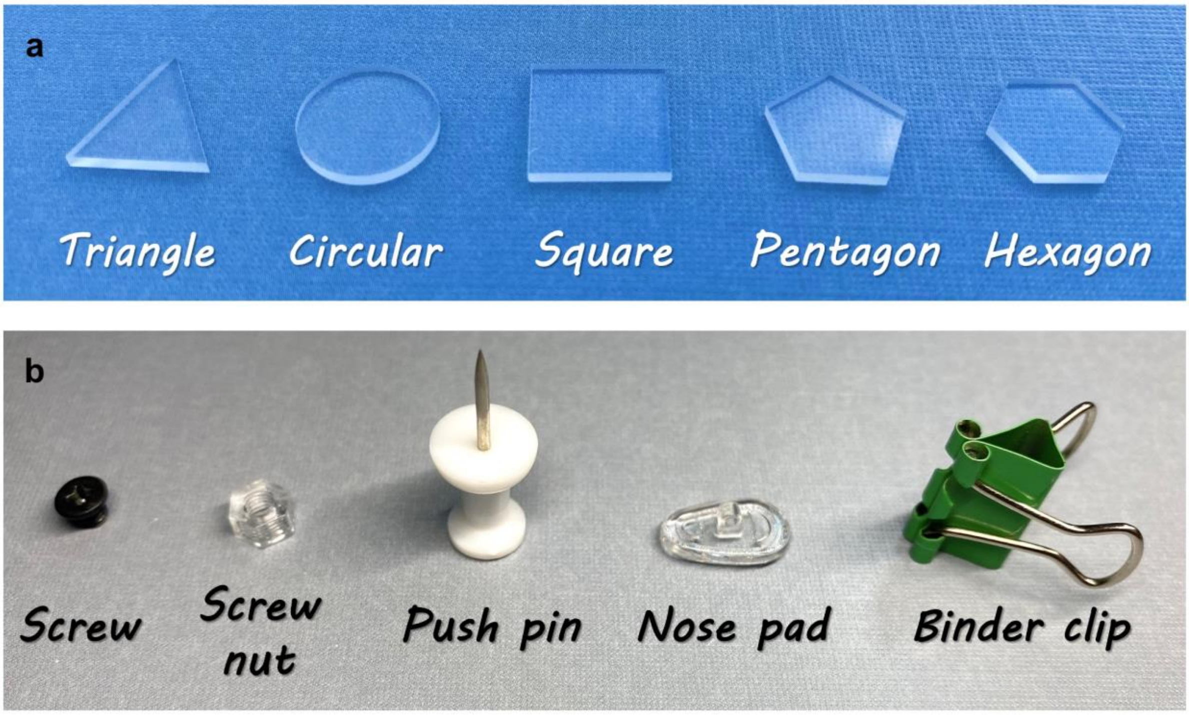

Figure S21. a) Photographs of 5 surface shapes (triangle, circular, square, pentagon, and hexagon). b) Photographs of 5 common objects (screw, screw nut, push pin, nose pad, and binder clip).

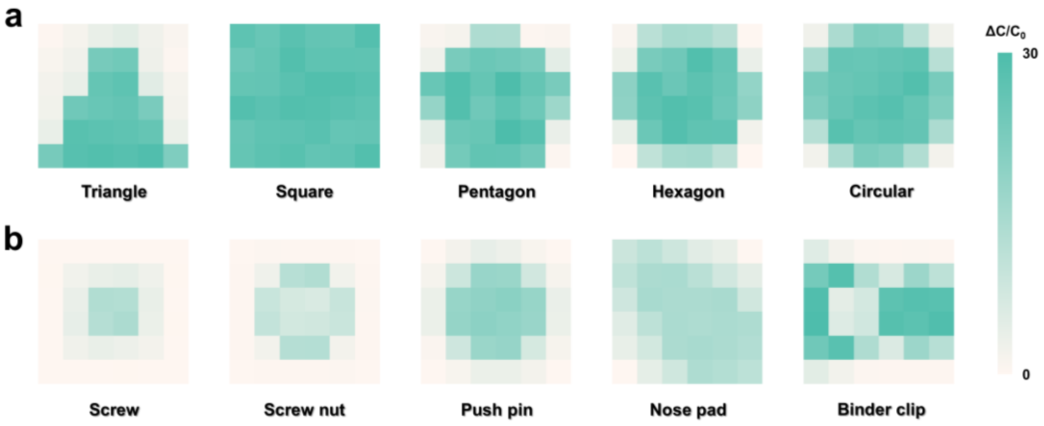

Figure S22. Capacitance response-pressure mapping (6 × 6 matrix) of a) 5 surface shapes and b) 5 common objects.

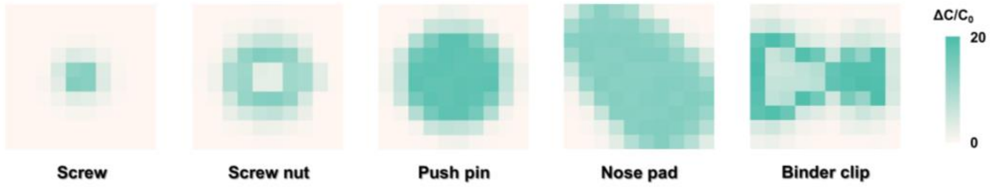

Figure S23. Capacitance response-pressure mapping (10 × 10 matrix) of 5 common objects.

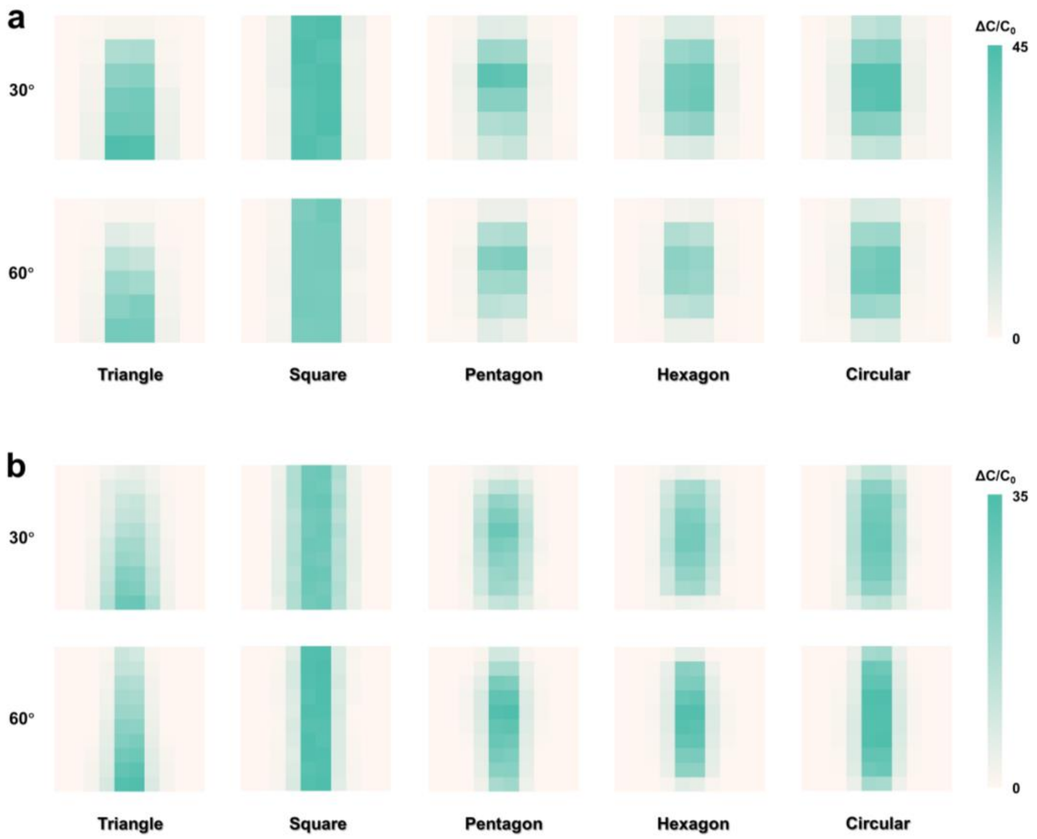

Figure S24. Capacitance response-pressure mapping of a) 6 × 6 matrix and b) 10 × 10 matrix based on 5 surface shapes at bending angles of 30° and 60°.

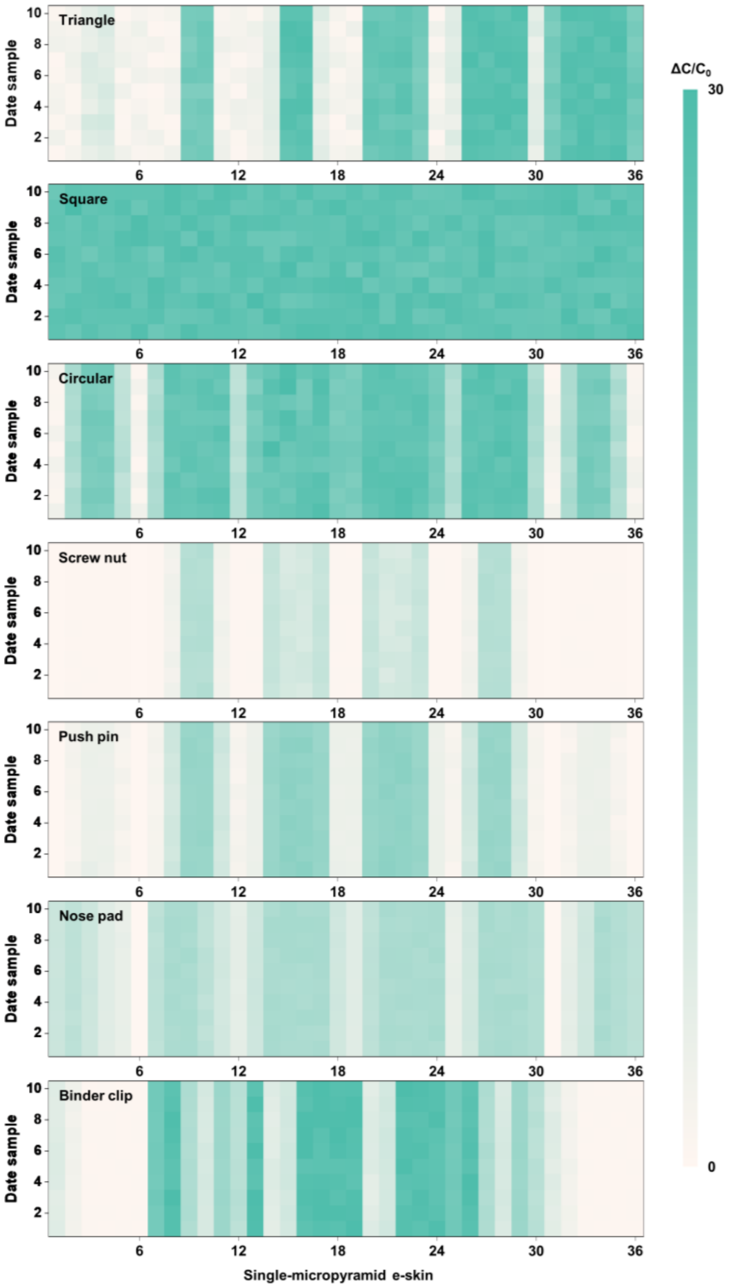

Figure S25. Distribution diagram of 10 sets of capacitance response-pressure mapping data collected by the triangle, square, circular, screw nut, push pin, nose pad, and binder clip.

Table S1. Sensing performance comparison of recently reported capacitive e-skin.

Ref.	Sensitivity [kPa^{-1}] (Max)	Response/ Recovery [ms]	LOD [Pa]	Compression Stability

[38]	0.15	6/6	0.35	10000
[39]	1.5	18/36	35	1500
[40]	4.5	50/50	0.2	500
[41]	11.8	15/15	0.2	5000
[42]	158.2	-/-	100	6000
[43]	171	5.6/5.6	1.2	20000
[44]	87.75	7.52/13.97	0.22	1000
[45]	365	30/60	1.78	10000
[46]	40.39	34/37	0.59	10000
[47]	4.4	16/46	0.8	50000
[48]	66.3	36/41	7	1000
This work	655.3	11.2/16.8	0.2	10000

Supplementary References

[1] K. Lichtenecker, *Phys. Z.* **1926**, 27, 115.

[2] Z.-M. Dang, J.-K. Yuan, J.-W. Zha, T. Zhou, S.-T. Li, G.-H. Hu, *Prog. Mater. Sci.* **2012**, 57, 660.
